# IPS e.max for All-Ceramic Restorations: Clinical Survival and Success Rates of Full-Coverage Crowns and Fixed Partial Dentures

**DOI:** 10.3390/ma12030462

**Published:** 2019-02-02

**Authors:** Silvia Brandt, Anna Winter, Hans-Christoph Lauer, Fritz Kollmar, Soo-Jeong Portscher-Kim, Georgios E. Romanos

**Affiliations:** 1 Department of Prosthodontics, Center for Dentistry and Oral Medicine (Carolinum), Johann Wolfgang Goethe University, Theodor-Stern-Kai 7, 60596 Frankfurt, Germany; h.c.lauer@em.uni-frankfurt.de (H.-C.L.); s.kim@med.uni-frankfurt.de (S.-J.P.-K.); 2 Department of Prosthodontics, Center for Dentistry, Oral Medicine, Julius Maximilian University, Pleicherwall 2, 97070 Würzburg, Germany; e-winter_a3@ukw.de; 3 Private Practice Dr. Fritz Kollmar, Friedrich-Ebert-Straße 55, 34117 Kassel, Germany; fitz@dreskollmar.de; 4 Department of Oral Surgery and Implant Dentistry, Center for Dentistry and Oral Medicine (Carolinum), Johann Wolfgang Goethe University, Theodor-Stern-Kai 7, 60596 Frankfurt, Germany; georgios.romanos@stonybrook.edu; 5 Germany and Department of Periodontology, School of Dental Medicine, Stony Brook University, Stony Brook, NY 11794, USA

**Keywords:** IPS e.max system, full-contour crown restorations, fixed partial dentures, survival rate, success rate

## Abstract

The IPS e.max system by Ivoclar Vivadent, offering a variety of products and indications, is widely used for all-ceramic restorations. We analyzed the clinical track record of these products in daily clinical practice, associating their restorative survival rate with various parameters to define recommendations for long-term stability. A total of 1058 full-coverage crowns and fixed partial dentures (FPDs) were evaluated retrospectively over up to 66.48 (37.05 ± 18.4) months. All were made of IPS e.max Press, IPS e.max CAD, IPS e.max Ceram or IPS e.max ZirPress and had been delivered by a private dental practice within three years. Uses not recommended by the manufacturer were also deliberately included. The five-year cumulative survival was 94.22% (i.e., 94.69% or 90.58% for glass-ceramic crowns or FDPs and 100% or 90.06% for zirconia-based crowns or FDPs). Significantly superior outcomes emerged for conventional vs. adhesive cementation and for vital vs. non-vital abutment teeth, but not for recommended vs. non-recommended uses. Caution is required in restoring non-vital teeth, but the spectrum of recommended uses should generally be reconsidered and expanded, given our finding of high survival and success rates for IPS e.max ceramics, even for uses not currently recommended by the manufacturer.

## 1. Introduction

Patients increasingly know about the availability of dental materials that are both esthetically pleasing and biocompatible [1,2]. All-ceramic restorations combine high biocompatibility with good optical and material properties [3], thus meeting both patient expectations and clinical requirements [4]. This is why this particular segment of the dental market has been growing for some decades [2]. All-ceramic restorations may be considered an evidence-based treatment modality, given a large number of available studies [5,6,7]. All-ceramic crowns have been shown in vitro to be a good alternative to metal-ceramic crowns [8] and clinical results to the same effect were provided by Etman and Woolford [9].

On closer examination, however, many studies have limitations such as narrow indications or small case numbers [10,11,12,13]. Another problem is strict inclusion and exclusion criteria, resulting in the overrepresentation of stable periodontal health and good oral hygiene [12,13,14,15,16,17] while turning a blind eye to cases involving temporomandibular dysfunction [11,12,13]. Hence, the qualifications that often get attached to the study of restorations have a way of removing the cases studied from daily clinical practice, where clinicians need to consider a variety of patient-specific risk factors whenever they plan and deliver dental restorations. Sometimes they conclude that pushing beyond the spectrum of indications for a given material is the way to go and, with the patient’s consent, will proceed to implement this plan.

There is a need for these clinicians to select from a multitude of ceramic materials that are intended for different indications, require different procedures and come with different recommendations for retention. IPS e.max (Ivoclar Vivadent, Ellwangen, Germany) is a lithium-disilicate system encompassing a comprehensive range of products for diverse uses and processing techniques. Being a glass-ceramic material, lithium silicate combines the advantages of permitting, although not requiring, adhesive luting for retention [18] in addition to offering maximum esthetics [10] and high fracture resistance [19]. Thanks to these benefits, it is a material widely used in clinical practice.

Given the increasing use of all-ceramic restorations in clinical practice, it is only reasonable to investigate them under real-life clinical conditions as well. To fill the aforementioned gaps left by the limiting inclusion and exclusion criteria of previous studies, we designed a retrospective investigation into the survival rates of all-ceramic IPS e.max restorations with no exclusion criteria applied, specifically including uses of the material not currently recommended by its manufacturer. Another goal was to analyze how the restorative failures involved were related to specific clinical parameters, thus helping to avoid errors and improve survival in daily clinical practice.

## 2. Materials and Methods

All restorations here evaluated had been delivered in a private practice in Germany between June 2011 and June 2014. The baseline totality of eligible restorations was 1101 full-coverage crowns and fixed partial dentures (FPDs) made from IPS e.max Press, IPS e.max CAD, IPS e.max Ceram or IPS e.max ZirPress (Ivoclar Vivadent). All these restorations met our requirement of having been fabricated in the same dental laboratory in accordance with the manufacturer’s instructions. For evaluation in this study, we additionally required that the patients complied with follow-up visits.

Given that 43 of these 1101 restorations were not followed up in the years to come, a total of 1058 restorations eventually met all inclusion criteria for evaluation. Since our intention was to evaluate the IPS e.max system under real-life conditions, we applied no exclusion criteria, thus specifically including uses of the material not currently recommended by its manufacturer. Table 1 gives an overview of how the various restorative products were distributed across the 1058 evaluable restorations.

For statistical analysis, general information (age, gender, anonymized patient ID) and additional treatment-related details were entered in spreadsheet software (Microsoft Excel). The patients were divided into different groups according to their age at the time of incorporation (≤ 30 years, 31–50 years, 51–70 years, ≥ 70 years). The parameters thus recorded included: abutment topography; type of support by implant and or natural abutment(s); vitality or non-vitality of natural abutments; nature of the opposing dentition; restorative design; restorative material; use for a recommended or non-recommended indication; luting technique; technician in charge of fabrication; interval from delivery to latest follow-up; as well as occurrence and management of complications and failures.

Any complete loss of a restoration was defined as a restorative failure influencing the survival curve of the time-to-event analysis. Kaplan–Meier probabilities of survival time could then be estimated, based on the number of failures documented throughout the observation period, for any of the crowns and FPDs considered. Aside from these survival rates, success rates were obtained separately by performing the respective Kaplan–Meier calculations based on all complications rather than on failures only. The aforementioned parameters were analyzed by log-rank testing for significant associations with restorative survival.

All statistical calculations were performed with BiAS (Biometric Analysis of Samples) software (rev. 11.05; Epsilon Verlag, Nordhastedt, Germany) and differences were considered significant at *p* ≤ 0.05. Approval for the study was obtained from the institutional review board (reference number: 17/2015).

## 3. Results

### 3.1. Baseline Data

The 1058 restorations meeting all inclusion criteria were evaluable over an observation period of up to 66.48 (37.05 ± 18.4) months. They included 922 single or splinted crowns and 136 FPDs in 368 (206 female and 162 male) patients aged 57.84 years at the time of delivery. Maxillary restorations accounted for 58.57% (540/922) of the crowns and 50.74% (69/136) of the FPDs. Adhesive cementation were used in 53.31% and conventional cementation in 46.69% of cases. Table 2 summarizes pertinent patient data, the distribution of the 1058 evaluable restorations by type (crowns or FDPs) and jaw (maxilla or mandible) and the cementation methods used.

### 3.2. Non-Recommended Uses and Restoration Subtypes

The manufacturer currently recommends IPS e.max Press and IPS e.max CAD for laminate/occlusal veneers, inlays/onlays, partial/full-coverage crowns, hybrid abutments/hybrid abutment crowns and for FPDs not extending beyond second premolars [20,21]. IPS e.max ZirPress and IPS e.max Ceram are used for framework veneering. Non-recommended uses accounted for 158 of the 1058 restorations here investigated, including FPDs supported by teeth (n = 32), implants (n = 9) or combinations of both (n = 2); splinted crowns supported by teeth (n = 101), implants (n = 9) or both (n = 4); and one tooth-supported single crown with a cantilever unit (n = 1). Table 3 gives a detailed listing of the restoration subtypes included in our evaluable overall sample of 1058 restorations.

### 3.3. Overall Success and Survival

A total of 35 restorations (3.3%) failed during the observation period, including 27 crowns and eight FDPs. The cumulative success rates were 97.36% (12 months), 96.32% (24 months), 90.37% (48 months) and 87.99% (60 months). Based on complete losses, the survival rates illustrated by the cumulative Kaplan–Meier curve were 98.83% (12 months), 98.41% (24 months), 96.93% (36 months), 95.52% (48 months) and 94.22% (60 months). Based on only the restorations used for non-recommended indications, five restorations (three crowns and two FPDs) had failed—all of these being failures concerning IPS e.max Press as a restorative material. The survival rate of non-recommended uses scarcely differed (and hence not significantly) from recommended uses (*p* = 0.85). Table 4 gives an overview of the five-year survival rates broken down by restoration types and the two major restorative materials involved.

### 3.4. Results for the Crown Restorations

**Figure 1:**
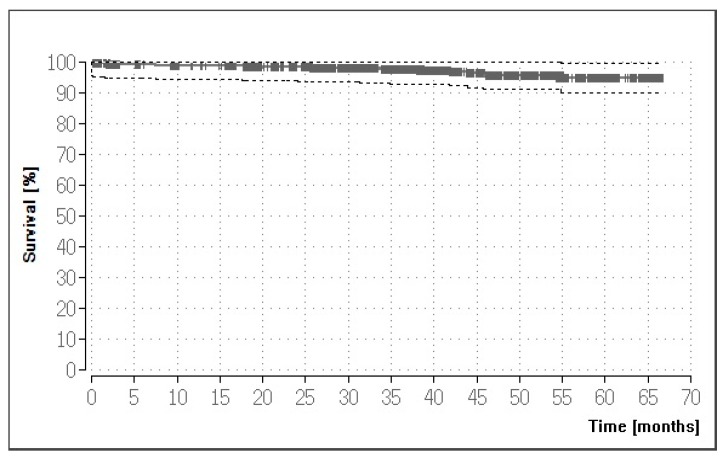
Kaplan–Meier survival curve for crown restorations.

Based on the 922 crown restorations, the survival rate was found to vary significantly with gender and age, being higher in male than female patients (log-rank test: *p* = 0.005) and within the age group of 31–50 years (log-rank test: *p* = 0.0043). Significant reductions in survival were seen for non-vitality compared to the vitality of abutment teeth (log-rank test: *p* = 0.0063) and for adhesive compared to conventional cementation (log-rank test: *p* = 0.0003). The 115 crowns used for non-recommended indications exhibited a survival rate almost identical to the crowns used for recommended indications (see Table 4). Figure 1 illustrates the Kaplan–Meier survival curve for all 922 crown restorations (five-year survival: 94.90%). Table 5 lists the different causes of all 27 failures.

### 3.5. Results for the FPD Restorations

**Figure 2:**
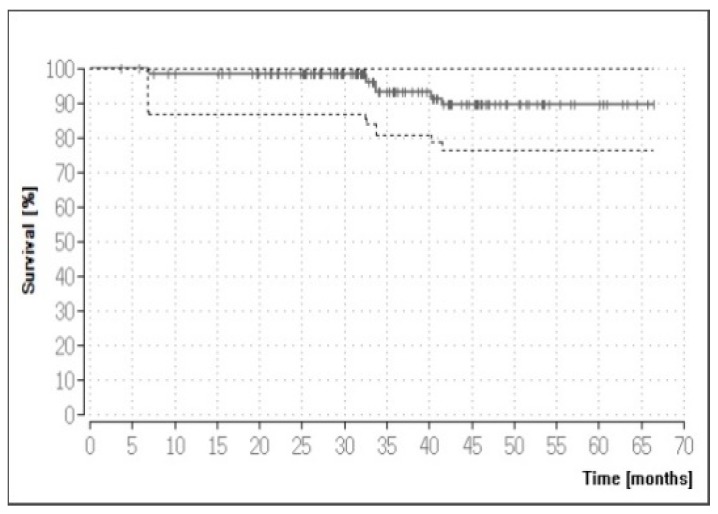
Kaplan–Meier survival curve for FPD restorations.

Based on the 136 FPDs, adhesive cementation was again found to reduce the survival rate compared to non-adhesive cementation (log-rank test: *p* = 0.0318). Unlike with the crown restorations, however, log-rank testing did not yield any significant differences for any of the other parameters based on FPDs. Non-recommended uses concerned 43 FPDs. They included the failure of two tooth-supported FPDs, but the resultant five-year Kaplan–Meier survival rate was actually (though not significantly) higher for the non-recommended than for the recommended uses (95.35% versus 88.40%; see Table 4). Figure 2 illustrates the Kaplan–Meier survival curve for the 136 FPDs (five-year survival: 89.44%). Table 6 lists the eight failures involved.

## 4. Discussion

Our results demonstrate the high survival and success rates of IPS e.max materials over up to 60 months. They do diverge somewhat from previous reports. Yang et al. [17], in a similar study design, reported a cumulative survival rate of 96.6% for 6855 restorations made of IPS e.max Press over five years. Part of the explanation for our different five-year survival of 94.22% might be that we investigated not only IPS e.max Press, but the whole IPS e.max range of products. Also, that previous group conducted their study under defined conditions linked to a military university hospital. The present study, by contrast, used patient files on record in a private practice. Given that no exclusion criteria were applied, more of these restorations were bound to be affected by patient-specific risk factors such as poor oral hygiene or periodontal problems. Also, we deliberately included uses of materials not currently recommended by the manufacturer.

In 2017, Rauch et al. [12] reported an 87.6% survival rate of lithium-disilicate single crowns after six years, which is lower than the rate presented here despite fewer risk factors due to defined exclusion criteria. One should, however, bear in mind the longer observation period in that study and its low case number of 25 crowns. These crowns were fabricated and inserted in both a private practice and a prosthetic university department, which incidentally yielded a (though not significantly) higher complication rate for the university-fabricated restorations. Our 100% survival rate for zirconia-based single crowns is consistent with another very high survival rate of 98.5% reported by Miura et al. [22]. Despite the provenance of our data (private practice) and our inclusion of non-recommended uses, our 90.58% survival rate for glass-ceramic FPDs is virtually identical to the 90.6% reported by Yang et al. [17], although both of these rates are lower than the 100% five-year survival for three-unit FPDs reported by Kern et al. [7], which again might be due to the different local scenarios involved.

Note, however, that all previous studies analyzed restorations conforming to the manufacturer’s recommendations. For example, Clausen et al. [23] investigated single crowns made of IPS e.max Press and Obermeier et al. [24] investigated implant-supported crowns made of IPS e.max CAD. The present study, by contrast, also includes 158 non-recommended uses. While five of these restorations failed (all of them made of IPS e.max Press), a significant difference between recommended and non-recommended uses was not observed. Despite this non-significant difference and the limited number of cases involved, it is nevertheless interesting to note that the non-recommended restorations actually showed higher survival rates. As a departure from previous studies, it is therefore fair to suggest that the range of indications listed by the manufacturer should be reconsidered and expanded where appropriate.

However, one point to consider in comparing our results to those of previous studies is that consistent definitions of ‘complication’ and ‘failure’ have often been lacking. Different causes of failure emerged. Technical complications included ceramic defects in the form of full-blown fractures or chipping. Events of this kind have been reported previously to constitute a frequent complication of all-ceramic single crowns, as evidenced in a 2017 review article by Aldegheishem et al. [25] and supported by Monaco et al. [8]. Consistent with statements by Reich et al. [13], we also noted failures resulting from endodontic complications such as hypersensitivity or apical osteitis, as well as from secondary caries of abutment teeth.

We also demonstrated a statistically significant association between survival rate and cementation type. Both conventional and adhesive cementation can be used with the IPS e.max system. Adhesive bonding is meant to increase the fracture resistance of restorations [26,27,28] but is highly technique-sensitive. Perfect drying/isolation [29] and appropriate etchability of the residual tooth structure are required. Also, the quality of the bond is amenable to surface conditioning, as has been demonstrated by applying different methods of cleaning to restorations after etching [30]. Although Kern et al. [7] and Esquivel-Upshaw et al. [31] observed no difference to this effect, conventional cementation emerged as superior in the present study. Again, one might implicate our different study design in this finding, considering that the restorations we analyzed were inserted in a private practice, where changing clinicians and practice-related circumstances may have played a role. Yet, on the flip side of this argument, Rauch et al. [12] reported in their study a higher complication rate for restorations delivered at a German university hospital than for those delivered by dentists in private practice.

Another statistically significant finding of the present study was the lower survival rate of crowns retained on non-vital than on vital teeth. Huettig et al. [32] and Fedorowicz et al. [33] previously reported a similarly increased rate of complications for non-vital abutment teeth, suspecting that these were biologically inferior to vital abutments. On another confirmatory note, Toman et al. [34] reported that the survival rate of crowns was significantly reduced on non-vital teeth and suggested that any restoration of non-vital teeth with lithium-disilicate crowns must be subject to rigorous case selection.

It should be considered that all restorations in our study had been fabricated from products of the same system (IPS e.max) and manufacturer (Ivoclar Vivadent). A comparable material by another manufacturer could not be included for comparison because no such material was available until very recently. This is over and above the fact that such a comparison would not have been readily feasible, given a clinical background of retrospectively evaluating data from a private practice. Similar study protocols in which specific materials were evaluated without comparison have been used previously [12,17]. Most importantly, the opportunity to assess one system as used in daily clinical practice across a wide spectrum of indications clearly outweighed the shortcoming that no different products were available for comparison.

The present study was designed as a non-experimental, analytical, retrospective cohort study on the basis of patient files archived in a private dental practice. Given this design, the correctness and completeness of the existing documentation had to be taken for granted. While the possibility of any issues in this regard should not be dismissed, this is a caveat inherent in most retrospective studies [35]. Given a large data base and a balanced distribution between maxillary and mandibular restorations, our approach did turn out to be appropriately suited to evaluating the clinical track record of the IPS e.max system and potential modifying factors.

As a case in point, the documentation available to us indicated for each restoration the dental laboratory where it had been fabricated. This allowed us to include only restorations known to have been fabricated by the same laboratory under the supervision of the same master dental technician, thus ensuring consistent fabrication standards, which can contribute greatly to favorable complication rates [36]. The detailed patient records also allowed us to consider the fabrication procedures in evaluating the outcomes. Previous reports on the survival rates of lithium-disilicate restorations were also based on standardized fabrication processes, but the samples they considered were heavily qualified [11,12,13,14,15,16,17]. Given our different study objective, which was to verify a real-life track record in daily clinical practice, we deliberately minimized criteria in compiling our sample. Also, our survival rates should be considered keeping in mind that any non-recommended uses of the studied material were specifically included.

Any complications that occurred were recorded during annual recall appointments, although the retrospective study design did imply very dissimilar follow-up times and observation periods, the latter ranging up to 66.48 months (mean 37.05 ± 18.4 months). While this may appear short compared with a follow-up time of up to 10 years in other studies, it has been shown repeatedly that most complications with IPS e.max restorations occur in the initial phase after insertion [16,17,32]. Hence, the observation period does seem to be adequately long for a conclusive evaluation of the material under study.

The present study produced a multitude of results. Thanks to a high case number and a wide spectrum of products and uses, we were able to address different questions that normally would have been addressed in separate studies. The fact that we deliberately refrained from exclusion criteria and used data that had accumulated in daily clinical practice enabled us to review the outcomes with IPS e.max products in real life. We could only achieve this by accepting risk factors such as bruxism, poor oral hygiene, periodontal problems, or non-recommended uses with their implications for long-term success. The high survival rates we obtained despite this background paints a favorable picture of the clinical track record of IPS e.max in everyday practice. The fact that these rates are highly consistent with the available literature on the subject underscores the conclusiveness of our findings. Also, a research focus on implant-borne IPS e.max restorations would be useful, given the increasing popularity of all-ceramic restorations for implants and that previous studies on the subject, while showing favorable material properties and survival rates, have covered limited samples or observation periods [37,38].

## 5. Conclusions

Within the limitations of this study, the use of IPS e.max may be recommended in clinical practice. Caution should be exercised in restoring non-vital teeth and in selecting luting techniques. High survival rates were obtained even for non-recommended uses of IPS e.max prompted by patient-specific considerations. Hence, the spectrum of indications for these products should generally be reconsidered and expanded where appropriate. Although we reviewed previous reports and filled some of the gaps they left, there is a need for further investigations similar in case number and design.

## Figures and Tables

**Table 1:** Distribution of restorative materials across the 1058 restorations.

Materials	n	Distribution
Total	1058	100.00%
IPS e.max Press	861	81.38%
Zirconia framework + IPS e.max ZirPress	87	8.22%
IPS e.max CAD	50	4.73%
Zirconia framework + IPS e.max Ceram	30	2.84%
IPS e.max Press + IPS e.max Ceram	27	2.55%
IPS e.max Ceram	3	0.28%

**Table 2:** Patient demographics, restoration types and cementation methods.

**Patients**		**Evaluable Restorations**	
Total (n)	368	Total (n)	1058
Female/male (n)	206/162	Full-coverage crowns (n)	922
Mean age (years)	57.84	Maxilla (n)	540
**Cementation**		Mandible (n)	382
Total (n)	1058	Fixed partial dentures (n)	136
Adhesive (n)	564	Maxilla (n)	69
Conventional (n)	494	Mandible (n)	67

**Table 3:** Detailed listing of the evaluable crowns and fixed partial dentures (FPDs).

Restorations	n	Distribution
All restorations	1058	100.00%
Tooth-supported single crowns	615	58.13%
Implant-supported single crowns	156	14.74%
Tooth-supported splinted crowns	126	11.91%
Tooth-supported FPDs	83	7.84%
Implant-supported FPDs	44	4.16%
Other ^1^	21	1.98%
Implant-supported splinted crowns	13	1.23%

^1^ For example, tooth/implant-supported or cantilever FPDs (9)/crowns (12).

**Table 4:** Five-year Kaplan–Meier survival rates.

Restorations	n	Survival
Cumulative (all restorations)	1058	94.22%
All full-coverage crowns	922	94.90%
Recommended uses	807	94.51%
Lithium-disilicate crowns ^1^	768	94.69%
Veneered zirconia-based crowns	39	100.00%
Non-recommended uses	115	94.95%
All fixed partial dentures	136	89.44%
Recommended uses	93	88.47%
Lithium-disilicate FDPs ^1^	43	90.58%
Veneered zirconia-based FDPs	50	90.06%
Non-recommended uses	43	95.35%

^1^ Made from IPS e.max products (CAD, IPS, Press with e.Ceram veneers or Ceram).

**Table 5:** Reasons for biological or technical failure of crown restorations.

Causes of Failure	n	Distribution
All full-coverage crowns	922	100.00%
Total number of failures	27	2.93%
Fracture of the restoration	5	0.54%
Apical osteitis	5	0.54%
Loss of retention	4	0.43%
Hypersensitivity	4	0.43%
Pre-prosthetic core fracture	3	0.33%
Chipping	2	0.22%
Root fracture	2	0.22%
Loss of implant	1	0.11%
Secondary caries	1	0.11%

**Table 6:** Reasons for biological or technical failure of FPD restorations.

Causes of Failure	n	Distribution
All fixed partial dentures (FPDs)	136	100.00%
Total number of failures	8	5.88%
Endodontic complications	3	2.21%
Ceramic chipping/fracture	2	1.47%
Root fracture	2	1.47%
Preprosthetic core fracture	1	0.74%
